# Low Frequency Microstimulation Is Locally Excitatory in Patients With Epilepsy

**DOI:** 10.3389/fncir.2018.00022

**Published:** 2018-04-04

**Authors:** Andrea Bartoli, Rémi Tyrand, Maria I. Vargas, Shahan Momjian, Colette Boëx

**Affiliations:** ^1^Department of Neurosurgery, Faculty of Medicine, Geneva University Hospitals, Geneva, Switzerland; ^2^Department of Neurology, Faculty of Medicine, Geneva University Hospitals, Geneva, Switzerland; ^3^Faculty of Medicine, University of Geneva, Geneva, Switzerland; ^4^Department of Neuroradiology, Faculty of Medicine, Geneva University Hospitals, Geneva, Switzerland

**Keywords:** DBS, epilepsy, microelectrode, microstimulation, safety, hippocampus

## Abstract

Deep brain stimulation (DBS) could become a palliative treatment for patients with drug-resistant epilepsy for which surgery cannot be proposed. The objective of this study was to perform microstimulation to measure the effects of DBS in epilepsy locally at the level of a few neurons, with microelectrode recordings, for the first time in patients with epilepsy. Microelectrode recordings were performed before, during and after microstimulation in nine patients with refractory epilepsy. Neuronal spikes were successfully extracted from multi-unit recordings with clustering in six out of seven patients during hippocampal and in one out of two patients during cortical dysplasia microstimulation (1 Hz, charge-balanced biphasic waveform, 60 μs/ph, 25 μA). The firing rates increased in four out of the six periods of microstimulation that could be analyzed. The firing rates were found higher than before microstimulation in all eight periods with increases reaching significance in six out of eight periods. Low-frequency microstimulation was hence sufficient to induce neuronal excitation lasting beyond the stimulation period. No inhibition was observed. This report presents the first evidence that microstimulation performed in epileptic patients produced locally neuronal excitation. Hence neuronal excitation is shown here as the local mechanism of action of DBS. This local excitation is in agreement with epileptogenic effects of low-frequency hippocampal macrostimulation.

## Introduction

Surgery is indicated for drug resistant epilepsy when the epileptogenic zone can be localized and when surgical removal is not related to unacceptable neurological or neuropsychological risks (Duncan, 2011). Alternatively, deep brain stimulation (DBS) is one of the palliative treatments for drug-resistant epilepsy in patients in which resective surgery cannot be proposed (Klinger and Mittal, 2016). The efficacy of DBS in reducing seizures, although encouraging, is variable and can be associated with side effects; determinants of its effectiveness have not been identified yet.

The development of DBS in epilepsy needs markers of its effects. Clinically, changes in seizure rates provide those. At the level of cerebral networks, intracerebral electroencephalograms do provide markers of DBS; in particular, these markers are provided through epileptic discharges (Goldberg and Coulter, 2013). Microelectrode recordings provide markers of the effects of DBS at the level of a few neurons (Alarcón et al., 2012). To better understand the effects of DBS at the level of a few neurons only, microstimulation was here applied with simultaneous microelectrode recordings.

Microstimulation was also motivated by the observation that DBS in patients with mesial temporal lobe epilepsy without hippocampal abnormalities can be successful with low amplitudes of stimulation, e.g., 0.5 V and 0.4 ms pulse duration (Boëx et al., 2011). This observation suggests that the efficacy of DBS can rely on the excitation of a small population of neurons provided that the stimulation location is optimal, contributing to the avoidance of side effects, such as visual or verbal memory decline that can occur with high amplitude hippocampal stimulation (Boëx et al., 2011; Miatton et al., 2011).

The term “microstimulation”, i.e., the application of small currents through microelectrodes, was introduced in 1968 with microelectrode stimulation performed to study pyramidal cell excitation in cats (Stoney et al., 1968). It has been since applied either for behavioral or for electrophysiological studies in animals (for a review see Bak et al., 1990; Histed et al., 2013). In humans microstimulation has been applied to visual cortex and it has been applied in the domain of DBS to study its mechanisms of action in the domain of movement disorders (Liu et al., 2012). In particular, a clear frequency-dependency of the firing rate has been shown during microstimulation of the globus pallidus in patients with dystonia; the average firing rate decreases as the frequency increases and is silenced at frequencies above 50 Hz (Liu et al., 2012). This result has been explained by short-term synaptic plasticity involving mainly GABAergic synapses; they could also be a consequence of the high charge injection levels that were applied leading to possible depolarization block.

Microstimulation in patients with epilepsy could bridge the existing gap between observations made *in vitro* with animal models and observations made in humans with macrostimulation.

Microstimulation was here applied with simultaneous microelectrode recordings to study locally the effects of DBS at the level of a few neurons only. Here, microstimulation was applied within safe charge injection limits to assess whether it is sufficient to modulate the neuronal activity of epileptic zones. Effects of *in vivo* microstimulation on the hippocampus and cortical dysplasia in refractory epilepsy patients are reported here for the first time.

## Materials and Methods

### Patients

Seven patients suffering from intractable epilepsy participated in the study. Intracranial invasive monitoring was indicated and offered because of the presence of conflicting scalp EEG data (except in patient H3 who directly underwent resective neurosurgery). Patients underwent stereotactic depth electrode implantation which positions were based on clinical findings, previous scalp EEG and imaging studies.

Five patients with temporal lobe abnormalities and two patients with cortical dysplasia participated. Neuronal activity could be identified in four patients with hippocampal microstimulation and in one patient with cortical dysplasia located in the anterior cingulum (Table 1). In the case of patient H3, microstimulation was performed under general anesthesia (Target-controlled infusion, TCI, Base Primea, Fresenius-Vial, Brezins, F; 3.2 μg.ml^−1^ propofol; Schnider et al., 1988, 1999; 0.3 ng.ml^−1^ sufentanil; Gepts et al., 1995).

**Table 1:** Features of patients.

	Age range at surgery	Age at onset of seizure (years)	Type of seizures	Cerebral abnormality
H1	[30–35]	9	Complex partial	Right hippocampal sclerosis
H2	[40–45]	17	Complex partial	Left hippocampal sclerosis
H3	[30–35]	20	Complex, secondarily generalized	Left hippocampal and parahippocampal dysplasia
H4	[50–55]	25	Complex, secondarily generalized	Right amygdala dysplasia
H5	[56–60]	2	Simple, secondarily generalized	Right extended mesial sclerosis
H6	[56–60]	25	Dyscognitive partial	Right hippocampal sclerosis
H7	[45–50]	2	Complex, secondarily generalized	Right hippocampal sclerosis
D1	[56–60]	7	Complex partial	Left anterior cingulate dysplasia
D2	[56–60]	7	Complex partial	Right frontal dysplasia

This work was conducted according to the ethical guidelines of the Declaration of Helsinki and was approved by the Ethical Committee of the University Hospitals of Geneva (CER 05-218, 14-076). Individually signed consent forms from patients were collected.

### Microelectrode Recordings and Stimulation

Microelectrodes, made each of eight microwires, were located at the tip of the macroelectrodes (WB09R-SP00×, Ad-Tech Instruments, Racine, WI, USA); macroelectrodes were all implanted stereotactically (each macroelectrode with eight macrocontacts, BF09R-SP05X). These platinum microelectrodes present an impedance of 1 MOhm, with a diameter of 40 μm.

Multi-Unit Activity was acquired using a 20 kHz sampling frequency recording system (Inomed Medizintechnik GmbH, Teningen, Germany; [150–3000Hz]). Noise threshold was fixed for comparison across all three conditions, i.e., before, during and after stimulation. Spike detection and clustering was realized with an algorithm introduced by Quiroga et al. (2004) and used in previous studies (Viskontas et al., 2009). The author RT visually inspected the clustering results.

The microstimulation was biphasic charge balanced pulses, cathodic first, 60 μs/phase, sent at a frequency of 1 Hz for a maximum of 2 min (NimEclipse System, Medtronic, Columbia Heights, MN, USA). The stimulation was monopolar, using one surface skin electrode as the return electrode (Neuroline ground, Ambu, Ballerup, Danemark). The voltage was adapted for a current target of 25 μA. Hence, the charge density did not exceed the accepted threshold of 150 μC/cm^2^/phase for platinum material, considering that microelectrodes were 40 μm in diameter (Merrill et al., 2005).

### Microelectrode Localization

A preoperative MRI was performed with a Siemens Trio 3.0T scanner using a 32-channel brain coil. The technical protocol was T2 FSE coronal (TR/TE 7520/114 ms, in-plane resolution 0.5 × 0.4 mm, slice thickness 3 mm), 3D T1 mp2rage (T/RTE 5000/2.89, in-plane resolution 1 × 1 mm, slice thickness 1 mm), DTI (TR/TE 8000/84, in-plane resolution 2 × 2 mm, slice thickness 2 mm, 30 diffusion directions), and 3D FLAIR (TR/TE 5000/419, in-plane resolution 0.9 × 0.9 mm, slice thickness 0.9 mm).

Postoperative CT was performed with a Siemens Somatom Definition Flash (Siemens, Erlangen, Germany). Slice thickness was 1.25 mm.

Preoperative high-resolution 3D sequences obtained at 3T and postoperative CT series were fused using commercially available software (Integrated Registration, AW Volume Share 5, GE Healthcare). Maximum intensity projections (MIP), multi-planar reformatting (MPR) and volume rendering (VR) were performed for better localization of the electrodes. Fused images illustrated location of microelectrode arrays (not performed for patient H3, H6 and H7 Supplementary Figure S1, Table 2).

**Table 2:** Spike frequency changes with microstimulation.

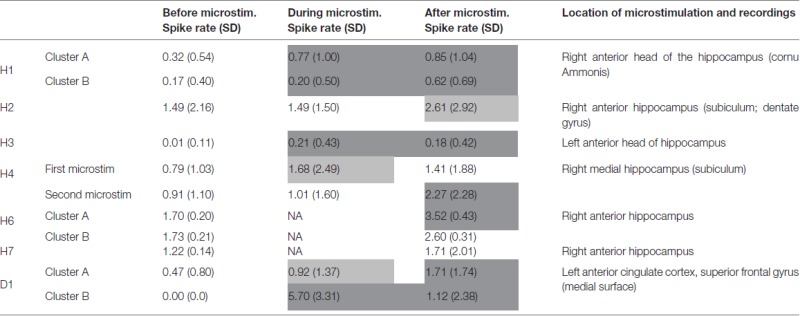

*Dark gray cells indicate significant increases in spike frequencies for every cluster compared to before microstimulation with a level of significance of *p* < 0.005; light gray cells indicate significant increases in spike frequencies compared to before microstimulation with level a significance of *p* < 0.05. Only one cluster was found, for patients H2, H3, H4 and H7*.

### Statistics

Analyses were performed using SigmaStat 3.11 (Systat Software Inc., Richmond, CA, USA). The differences in firing rates, described by the number of spikes per second, for every different condition before, during and after stimulation, were assessed by a Mann-Whitney Rank Sum Test, their distributions being not normal. Every different period, i.e., before, during and after, lasted at least for 1 min; specifically, at least 60 data points were collected per period. In addition group comparisons were performed with non parametric Friedman test comparing periods of exactly 60 s, as exact sample sizes are required for this statistic test. The first 60 s of the stimulation periods were compared to the latest 60 s of the pre-stimulation periods. The first 60 s of the post stimulation periods were compared to the latest 60 s of the stimulation periods.

## Results

Microstimulation performed within the hippocampus and within a cortical dysplasia of the anterior cingulum induced increases in spike frequencies during microstimulation, reaching significant levels in four out of the six stimulation periods that could be analyzed; Table 2). These increases lasted beyond the stimulation *per se* for all stimulation periods, reaching significant levels in six out of eight stimulation periods (Mann-Whitney Rank Sum Test, Table 2). No decrease in spike frequencies was observed. Group comparison performed for all five patients for who stimulation periods could be analyzed, indicated again significant increases in spike frequencies with microstimulation (χ^2^ = 38.61, *p* < 0.001; Friedman test). Group comparison between spike frequencies obtained right before stimulation and right after stimulation, performed for all seven patients, indicated again significant increases in spike frequencies with microstimulation lasting after stimulation (χ^*2*^ = 165.8, *p* < 0.001; Friedman test).

**Figure 1:**
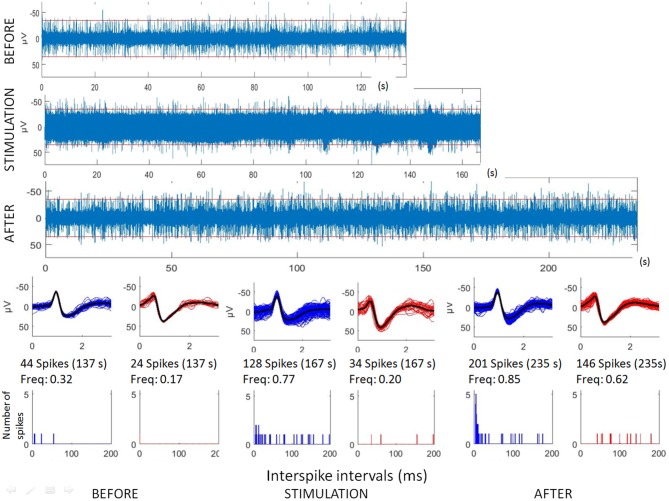
Analyses of hippocampal microstimulation (patient H1, 1 Hz). Top: raw microelectrode recordings (before, during and after stimulation; [150–3000Hz]). Middle: spike waveforms obtained with clustering of microelectrode recordings (number of spikes, duration of the recordings, frequencies of spike occurrences—Freq). Bottom: interspike interval histograms (ordinate number of times that the delay between two consecutive spikes is within the category given in abscissa, with categories of 1 ms. Note that all spikes were not found with delay between two consecutive spikes lower than 200 ms.

**Figure 2:**
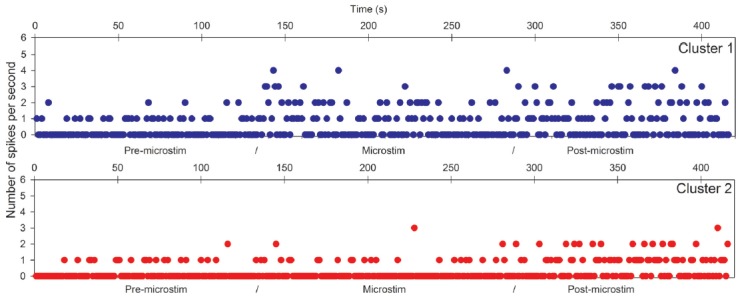
Hippocampal microstimulation (patient H1, 1 Hz). Effects of microstimulation on spike frequencies illustrated with histograms of spike occurrences during the whole recording (ordinate: number of spikes per second, before, during and after microstimulation; time indicated in abscissa).

**Figure 3:**
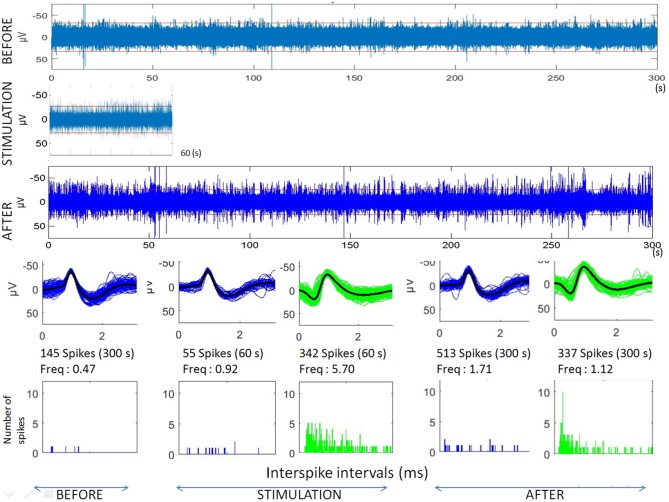
Analyses of microstimulation within a cortical dysplagia (patient D1, 1 Hz). Top: raw microelectrode recordings before, during and after microstimulation; [150–3000Hz]). Middle: spike waveforms obtained with clustering of microelectrode recordings (with number of spikes, duration of the recordings, frequencies of spike occurrences—Freq—and their standard deviations). Bottom: interspike interval histograms (ordinate number of times that the delay between two consecutive spikes is within the category given in abscissa, with categories of 1 ms).

**Figure 4:**
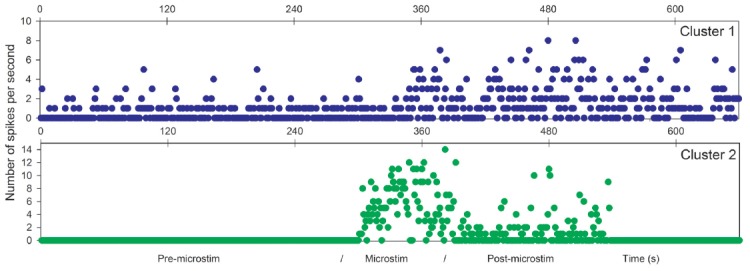
Microstimulation within a cortical dysplasia (patient D1, 1 Hz). Effects of microstimulation on spike frequencies illustrated with histograms of spike occurrences during the whole recording (ordinate: number of spikes per second, before, during and after microstimulation; time indicated in abscissa).

Raw recordings and clustering of spikes recorded before, during and after microstimulation periods are presented in Figures 1, 3, respectively, for the first two patients H1 and D1 as examples. The changes in firing rates are illustrated in Figures 2, 4 for the same patients.

The first lines of Figures 1, 3 are the raw signals of microelectrodes recorded before stimulation, the second lines are the signals recorded during stimulation, and the third lines are the signals recorded after the microstimulation periods. Spike clusterings indicated two different types of cells in both of these examples. The number of their occurrences and frequencies are indicated for the three different periods, i.e., before, during and after microstimulation. Note the increases of their occurrences during and after microstimulation; in particular, one new cell appeared with cortical microstimulation (Figures 2, 4).

Note that no seizures occurred for 12 h following the microstimulation of all the patients of the group, suggesting microstimulation did not favor seizures.

## Discussion

Low-frequency microstimulation of hippocampus or of cortical dysplasia was demonstrated as sufficient to increase spike frequencies, exciting locally the neuronal activity involved in medically intractable epilepsy. As demonstrated in rats and mice, microstimulation predominantly activates axons and causes a sparse pattern of activation in a small volume around the microelectrode tip (Histed et al., 2007).

### Hippocampus Stimulation

The excitation induced by hippocampal low-frequency microstimulation, head or subiculum, supports the epileptogenic effect of low-frequency macrostimulation at this anatomical target (Boëx et al., 2007). Specifically, low-frequency macrostimulation (5 Hz) of the hippocampus resulted in an increase of interictal epileptiform discharges that can potentially generate seizures. In agreement with this literature, the present study suggests that the epileptogenic effect of low-frequency hippocampal macrostimulation observed in patients with epilepsy could be related to excitation at the neuronal level. A possible explanation could be a glutamaergic release induced by stimulation in agreement with principal hippocampal cell types (Cavus et al., 2016). Because of the low proportion of hippocampal GABAergic cells, these cells need to be specifically targeted to produce inhibition of the hippocampus as performed with optogenetic studies of the hippocampus (Krook-Magnuson et al., 2013).

If low-frequency hippocampal macrostimulation (5 Hz) or microstimulation (1 Hz) are both consistent with an epileptogenic effect in patients, this seems in contradiction with a decrease in neuronal activity reported with other hippocampal low-frequency stimulations performed in patients with epilepsy shown to be efficient in reducing interictal discharges (Alarcón et al., 2012; during 0.1 Hz, 1 ms, 6–8 mA, monophasic square pulse). Studies performed in kindled rats, supported inhibition or depression effects of low frequency direct hippocampal stimulation (Mohammad-Zadeh et al., 2007; Zhang et al., 2009; both monophasic square pulses). Nevertheless, within these studies, the applied stimuli differ markedly from the stimuli used in the present group of patients. Stimuli applied in all chronic DBS studies performed in human are all charge-balanced pulses, such as pseudo-monophasic (e.g., Medtronic stimulators, Medtronic Inc., Minneapolis, MN, USA; Boston Scientific stimulators, Boston Scientific, Marlborough, MA, USA; St. Jude Medical, St. Paul, MN, USA) or biphasic pulses (e.g., the present study) to avoid lesion in the neural tissue (Lilly et al., 1955; Mortimer et al., 1970). Indeed, no charge-balanced stimuli, such as monophasic-square pulses can create damage to the tissue because of the resulting products of non-reversible electrochemical reactions as known and applied in human safe electrical protocols (Merrill et al., 2005). As a consequence, lesions of the surrounding cells can contribute to produce inhibition of neuronal activity (Piallat et al., 2009) and contributed to overestimating the inhibitory effects reported in animal studies using low-frequency stimulation (Brummer and Turner, 1977).

While low frequency stimulation of the head of the hippocampus or of the subiculum does not appear to be anti-epileptogenic (Boëx et al., 2007), ventral hippocampal commissure (Kile et al., 2010; Rashid et al., 2011) or fornix (Koubeissi et al., 2013) stimulation could be anti-epileptogenic. Local neuronal excitation can have different effects on the networks to which these neurons are part of. They are certainly different between hippocampal *per se* or hippocampal commissural stimulations.

### Cortical Dysplasia Microstimulation

Cortical dysplasia is also a common cause of medically intractable epilepsy in both children and adults (Goldberg and Coulter, 2013). In patient D1, microstimulation of the cortical dysplasia led to a sustained increase of the firing rates. Hence, microstimulation can also modulate neocortical neuronal activity. Changes in cortical firing rate have been suggested as a means of seizure prevention (Truccolo et al., 2011), as supported by long-term low-frequency macrostimulation that has been shown to be anti-epileptogenic in patients with cortical epileptic focus (Matsumoto et al., 2005; Elisevich et al., 2006; Yamamoto et al., 2006; Hsu et al., 2011). The mechanism underlying the increase in firing rate with low-frequency stimulation can be found in an increased cortical excitability resulting from a lack of inhibitory interneurons. Indeed, experimental models of cortical dysplasia suggest a decrease of inhibitory interneurons as one of the main causes of epileptogenesis with an imbalance between excitatory and inhibitory activity (models obtained by irradiating rats *in utero*, Calcagnotto et al., 2005) and an alteration in firing rates and patterns of these interneurons (Zhou and Roper, 2011).

Studies of cortical microstimulation in monkeys have been described. High-frequency microstimulation can produce what has been recently described as “neural hijiacking” where the electrical stimulation eliminates and replaces the ongoing natural activity without summation, again demonstrating the possibility of cortical neural excitation with microstimulation (Griffin et al., 2011; Cheney et al., 2013).

In conclusion, microstimulation effects on microelectrode recordings were here studied for the first time in patients with epilepsy. Low-frequency microstimulation of the hippocampus or of cortical dysplasia was demonstrated as sufficient to increase spike frequencies, exciting the neuronal activity of cerebral areas involved in refractory epilepsy. In agreement with studies applying realistic stimuli, local neuronal excitation appears as the principal local mechanism of action of safe DBS. The effects of this local neuronal excitation are then dependent principally of the cerebral networks these neurons are involved and on the frequency of the stimulation, which were not studied here.

## Author Contributions

AB: interpretation of data, drafting, revising, final approval, agreement to be accountable for all aspects of the work in ensuring that questions related to the accuracy or integrity of any part of the work are appropriately investigated and resolved; RT: acquisition, analysis, interpretation of data, drafting, final approval, agreement to be accountable for all aspects of the work in ensuring that questions related to the accuracy or integrity of any part of the work are appropriately investigated and resolved; MIV: acquisition, analysis, interpretation of data, drafting, revising, final approval, agreement to be accountable for all aspects of the work in ensuring that questions related to the accuracy or integrity of any part of the work are appropriately investigated and resolved. SM: acquisition, revising, final approval, agreement to be accountable for all aspects of the work in ensuring that questions related to the accuracy or integrity of any part of the work are appropriately investigated and resolved; CB: conception, acquisition, analysis, interpretation of data, drafting, revising, final approval, agreement to be accountable for all aspects of the work in ensuring that questions related to the accuracy or integrity of any part of the work are appropriately investigated and resolved.

## Conflict of Interest Statement

The authors declare that the research was conducted in the absence of any commercial or financial relationships that could be construed as a potential conflict of interest.

## Supplementary Material

FIGURE S1 Preoperative high-resolution MRI fused with postoperative CT.
