# Supplementary figures and images for: Low Frequency Microstimulation Is Locally Excitatory in Patients With Epilepsy
Source: Front Neural Circuits. 2018 Apr 4;12:22. doi: 10.3389/fncir.2018.00022 (PMC5893788; doi:10.3389/fncir.2018.00022)

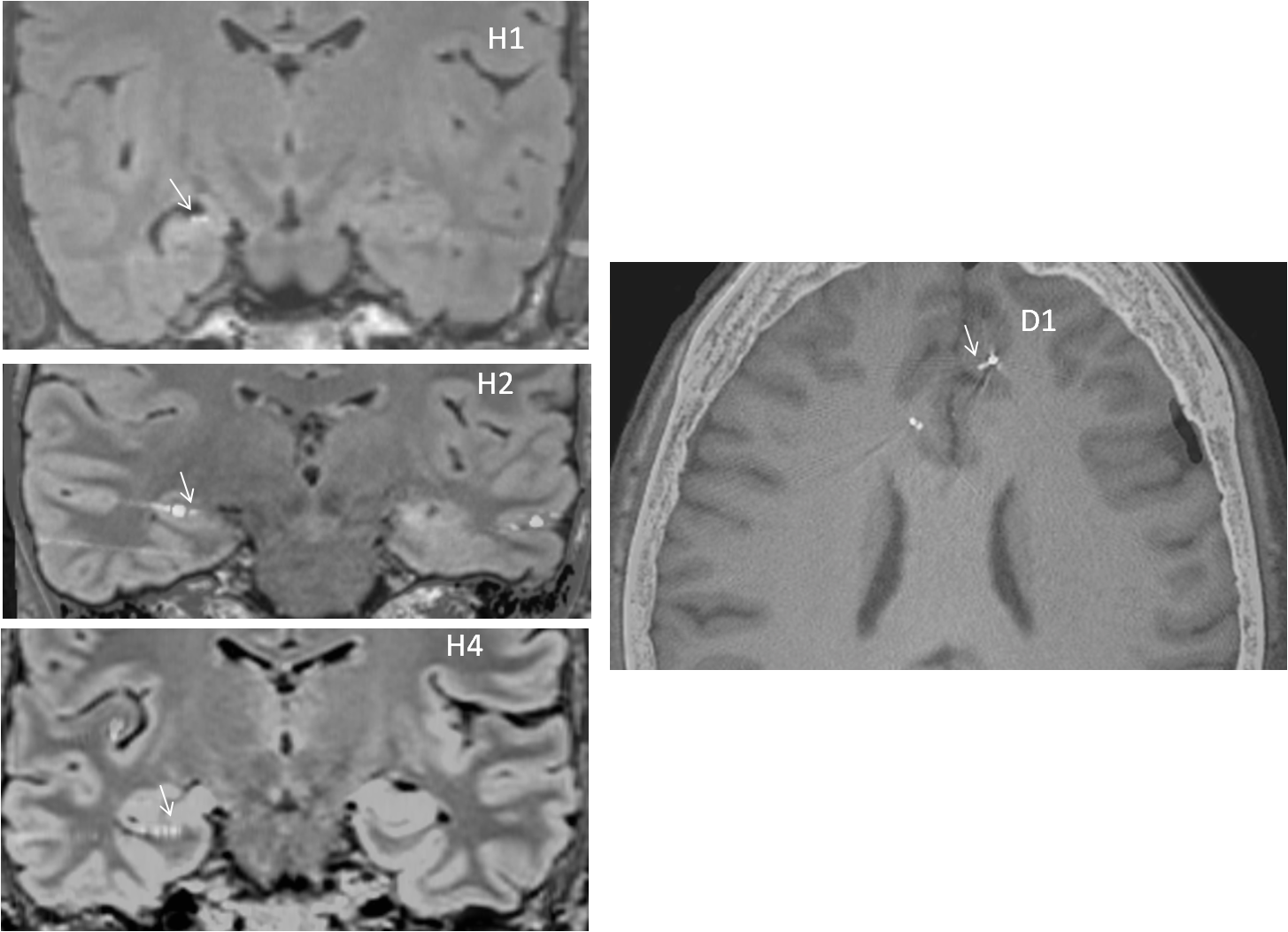

Supplement: FIGURE S1 — Preoperative high-resolution MRI fused with postoperative CT. [file Image_1.TIF]
